# ﻿Philippine herpetology (Amphibia, Reptilia), 20 years on: two decades of progress towards an increasingly collaborative, equitable, and inclusive approach to the study of the archipelago’s amphibians and reptiles

**DOI:** 10.3897/zookeys.1190.109586

**Published:** 2024-01-30

**Authors:** Camila G. Meneses, Kier Mitchel E. Pitogo, Christian E. Supsup, Rafe M. Brown

**Affiliations:** 1 Department of Ecology and Evolutionary Biology and Biodiversity Institute, University of Kansas, Lawrence, Kansas 66045, USA University of Kansas Lawrence United States of America

**Keywords:** Biodiversity, conservation, distribution, Southeast Asia, systematics, taxonomy

## Abstract

A first review of the history, status, and prospects for Philippine herpetology conducted more than two decades ago (2002) summarized the diverse topics studied and highlighted the development and achievements in research up to the year 2000. This study revisits and re-assesses what Philippine herpetology has accomplished, both as a discipline and a community, during the last two decades (2002–2022). A total of 423 herpetological publications was collated, revealing a substantial increase in annual publications, rising from approximately four per year during 2002–2008 to around 28 per year in 2009–2022. Half of the published studies focused on squamate reptiles (lizards 30.5%, snakes 21%) and 28.4% on amphibians, 5.9% on turtles, and 2.6% on crocodiles. The remaining 11.6% of studies focused simultaneously on multiple taxa (i.e., faunal inventories). Diversity and distribution (35.2%) and ecological (26.5%) studies remained popular, while studies on taxonomy (14.9%), phylogenetics and biogeography (11.8%), and conservation (11.6%) all increased. However, geographical gaps persist urging immediate surveys in many understudied regions of the country. Finally, we found a balanced representation between Filipino and foreign first authors (1.0:1.1), yet a substantial gender gap exists between male and female first authors (7.1:1.0). Nonetheless, the steep increase in publications and the diversity of people engaged in Philippine herpetology is a remarkable positive finding compared to the 20 years preceding the last review (1980–2000). Our hope is that the next decades will bring increasingly equitable, internationally collaborative, and broadly inclusive engagement in the study of amphibians and reptiles in the Philippines.

## ﻿Introduction

Sustained, regionally focused, field-based research programs focusing on the ecology and evolution of amphibians and reptiles provide insight into many conceptually intriguing, unique, and fundamental questions relating to the origin, accumulation, and continued survival of Earth’s biodiversity (Brown and Alcala 1970a; Brown et al. 2013a; Gainsbury and Meiri 2017; Greenbеrg et al. 2018; Meiri et al. 2020; Sheu et al. 2020; Zimin et al. 2022; Camaiti et al. 2023). Regionally oriented research by herpetological systematists, biogeographers, and macroecologists have elevated our understanding of the global phylogenetic and evolutionary underpinnings of amphibian and reptile biodiversity by relating the distribution of this diversity to the geographical template itself (Esselstyn et al. 2010; Böhm et al. 2013; Barley et al. 2014, 2015; Roll et al. 2017; Vidan et al. 2019; Gumbs et al. 2020; Bernstein et al. 2021a). Similarly, regional studies on topics ranging from natural histories of species from particular areas to global analyses of clade-wide trait variation (Vidan et al. 2019; Camaiti et al. 2022) and organismal genomic variation (Formenti et al. 2022; Chan et al. 2022; Hutter et al. 2022) now contribute significantly to our collective understanding of conservation challenges facing highly imperiled amphibian and reptile populations (Böhm et al. 2013; González-del-Pliego et al. 2019; Tingley et al. 2019; Gumbs et al. 2020; Chapple et al. 2021; Cox et al. 2022; McDonald et al. 2022; Womack et al. 2022; Slavenko et al. 2023).

**Figure 1.**
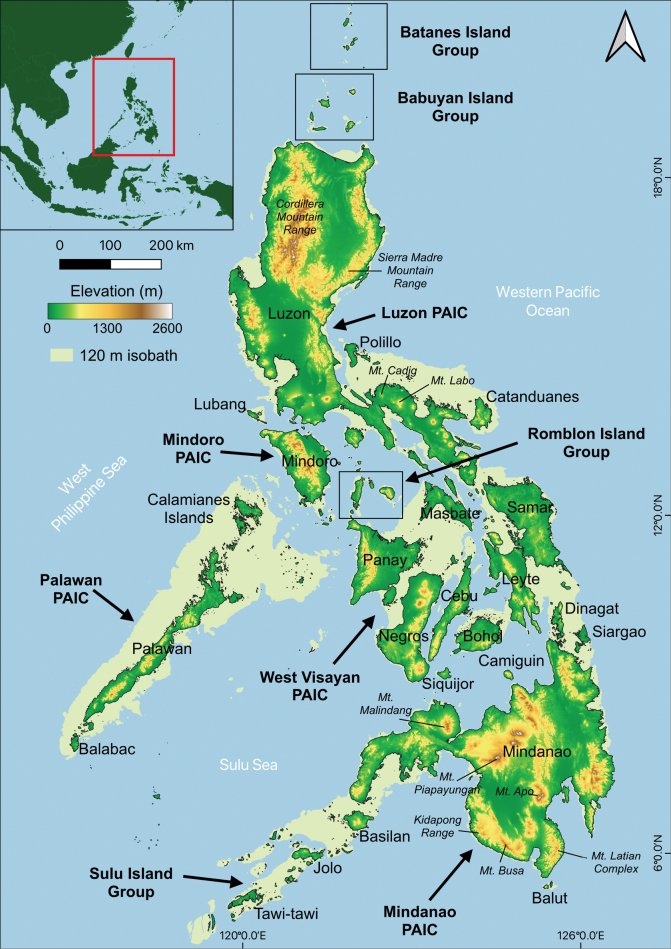
Map of the Philippine archipelago, situated in Southeast Asia (inset map), showing the recognized Pleistocene Aggregate Island Complexes (PAICs) and small island groups.

The Philippine Archipelago, which has been the focus of sustained herpetological inquiry for more than a century (Taylor 1921, 1922a, b, 1928; Leviton 1963a; Brown and Alcala 1970a, 1978, 1980; Brown et al. 2002a, 2012b, 2013a; Diesmos and Brown 2011; Diesmos et al. 2014, 2015; Leviton et al. 2014, 2018), is situated adjacent to the Southeast Asian mainland, between the Western Philippine Sea and the Western Pacific Ocean (Fig. 1). This unique archipelago is home to numerous remarkable evolutionary radiations of amphibians and reptiles (Brown and Guttman 2002; Brown et al. 2002a, 2009a, b, 2013a, 2015a; Evans et al. 2003; Brown and Diesmos 2009; Siler and Brown 2010, 2011; Siler et al. 2010a, b, c, d, 2011a, b, c, 2013, 2014a, b, c; Welton et al. 2013a; 2014a, b; Weinell and Brown 2017; Weinell et al. 2020a, b, [Bibr B277][Bibr B94], 2022; Flury et al. 2021), many of which have only recently been characterized, and some of which remain to be studied in depth (e.g., Brown et al. 2010a, 2011a, b, 2012a; Linkem et al. 2010a, b, c; Linkem and Brown 2013; Wynn et al. 2016; Oliver et al. 2018; Davis et al. 2020; Bernstein et al. 2021b; Eliades et al. 2021). Currently, there are approximately 475 recognized species of amphibians and reptiles in the Philippines, 76.2% of which are endemic, and most of which can be characterized as (1) geographically circumscribed species, whose distributions coincide with one of the archipelago’s 5–7 major faunal regions (Brown and Alcala 1970a, 1978, 1980; Brown et al. 2017); (2) range-restricted species from isolated islands (e.g., Brown and Alcala 1974; Brown et al. 1997; Ferner et al. 2001; Linkem et al. 2010a; Oliveros et al. 2011; Brown et al. 2018; Meneses et al. 2022), or (3) species limited to geologically isolated and/or upland habitats (e.g., Brown and Alcala 1961, 1970b, 1982a; Ferner et al. 1997; Brown et al. 1999a, b, 2020; Linkem et al. 2010b; Siler at al. 2010c). However, this estimate is expected to change over time with ongoing biodiversity inventories, taxonomic revisionary studies, critical reappraisals of earlier works, and the novel application of technologies (e.g., genomic data, ecological niche modeling) still emerging today (Brown et al. 2002a; Brown 2006; Brown and Siler 2013; Diesmos et al. 2015).

Just more than two decades ago, Brown et al. (2002a) conducted a comprehensive review of Philippine herpetological studies. The authors summarized the history of amphibian and reptile studies and included the distinct periods which characterized the development of herpetological research in the country, highlighting the important contributions of biologists during the last decades leading up to the turn of the century. That review centered on five topical themes or areas of research focus: (1) diversity and distribution, (2) taxonomy, (3) ecology, (4) phylogenetic systematics and biogeography, and (5) conservation. As a result of that exercise, it became abundantly clear that most of the archipelago’s earlier herpetological studies (prior to the 2000s) were predominantly focused on species diversity, taxonomy, and biogeography (and, to a lesser extent, ecology, and conservation). Despite the many papers focused on single species descriptions, but also including synthetic reviews (e.g., Brown and Alcala 1978, 1980), an immense amount of taxonomic work was still needed by the early 2000s. This is because the majority of studies to that date were descriptive, based solely on traditional morphological characters (i.e., measurements, meristic data like scale counts, and comparisons of discrete character states), and only selected clades had been comprehensively reviewed with the goal of synthetic considerations of those particular faunal groups (Taylor 1921, 1922a, b; Inger 1954; Leviton and Brown 1958; Leviton 1962, 1963a, b, 1964a, b, c, d, 1965a, b, 1967, 1968, 1979, 1983; Brown and Alcala 1974, 1980, 1994). As discussed by Brown (2006, 2007) and Brown and Stuart (2012), the use of multiple data streams and integrative approaches (including phylogeny) for more robust, pluralistic, and quantitative approaches to species recognition was just in their infancy (Brown and Diesmos 2002; Brown and Guttman 2002; Diesmos et al. 2002; Brown et al. 2003).

Brown et al. (2002a) also highlighted some of the gaps in other research areas. For instance, on the subject of biogeography and conservation, they emphasized how understanding patterns of Philippine amphibian and reptile distributions would be essential to formulating effective conservation and management strategies (Brown and Alcala 1961, 1986; Alcala and Custodio 1995; Diesmos et al. 2002). However, at the time of their review, Brown et al. (2002a) emphasized that the full informative potential of a comprehensive understanding of verified species distributions had not been fully realized due to limited information from many unexplored islands groups (i.e., the Batanes, Bubuyans, Lubang, Leyte, Masbate, Mindoro, the Romblon Island Group, Siquijor, the Sulu Archipelago, and Samar), as well as numerous high-elevation mountain ranges of the archipelago’s largest islands, Luzon and Mindanao. At that time (Brown et al. 2002a), documentation of patterns of species occurrences and community structure along elevational gradients was just beginning to take shape (Brown and Alcala 1961; Custodio 1986; Alcala and Custodio 1995; Alcala et al. 1995; Brown et al. 1996, 2000a; Heaney et al. 2000). Numerous other topics were highlighted, including formative areas of research that had become a focus by mid-century, but for which no follow-up investigations had been forthcoming during the last 20–40 years (Brown et al. 2002a). These included studies of reproductive biology (Alcala and Brown 1956, 1982; Alcala 1962; Brown and Alcala 1982b), physiology (Alcala and Brown 1966), development (Alcala 1962; Alcala and Brown 1982), and basic population biology and demography (Alcala 1967, 1970; Alcala and Brown 1967).

In general, Brown et al. (2002a) emphasized that these conspicuous gaps in Philippine herpetological research would most likely be addressed most effectively through collaborative efforts of teams of institutions (universities, local government units, non-governmental organizations, stakeholder communities) and the participation of diverse groups of foreign and local researchers, students, and local community representatives. Ultimately, the results accessible via open-access peer-reviewed publications–such studies reinforced by integrative analyses of multiple data types that are freely accessible through web-based platforms (e.g., HerpNet, VertNet, etc.) and specimen-associated data provided by natural history museums–could be harnessed in such a way that a more inclusive, transparent, broadly participatory future of Philippine herpetology could be realized (e.g., Brown et al. 2012b, 2013b). With the turn of the millennium and given the inevitable shift in herpetological research that was coming, Brown et al. (2002a) reviewed and took stock of the accomplishments, discoveries, strengths, and shortcomings of what they envisioned as a global community of herpetologists interested in Philippine biodiversity. Today, we revisit that same general topic, but we do so with the sense that another, pronounced, and unique period of the historical development of herpetology in the Philippines is coming in the years ahead.

In this paper, we revisit studies conducted from 2002–2022 to re-assess the state of Philippine herpetological research, 20 years after the review of Brown et al. (2002a). We summarized the last two decades of published studies in five general areas or topical themes, as discussed in the previous review. One of our goals was to explore whether these studies addressed gaps previously identified (Brown et al. 2002a). Trends during the last two decades of taxonomic studies were examined, including attention to data types (e.g., morphology versus molecular data), modern quantitative methodology (e.g., phylogenetics, advanced statistical procedures, quantitative biogeographical inference), and how these were integrated or used in statistical species delimitation (e.g., Barley et al. 2013; Welton et al. 2013a). We also sought to obtain an explicitly geographical or spatial overview of the last twenty years of research and identify priority areas that receive little attention and now represent challenges for the future. Finally, to determine who is primarily involved in Philippine herpetological research–and to critically and transparently address the question of diversity, equity, and inclusion in our field (Have we broadened engagement? Is our community more diverse now than it was a quarter century ago?)–we classified published studies by the lead author’s nationality and gender and explored the diversity of people who study Philippine herpetology.

## ﻿Methods

### ﻿Literature review

We employed a systematic literature review of Philippine herpetology published from 2002–2022, following the guidelines from the updated Preferred Reporting Items for Systematic Reviews and Meta-Analyses (PRISMA) statement (Page et al. 2021). We compiled a dataset of peer-reviewed journal articles by searching through Google Scholar and Web of Science. The following keywords were used: “Philippine amphibians,” “Philippine reptiles,” “Philippine lizards,” “Philippine snakes,” and “Philippine anurans/frogs.” The list of papers compiled was manually supplemented to include articles not captured in the preliminary search. This includes articles addressing other areas or topics, but which included substantial Philippine specimens (or data derived from Philippine studies, and now available in the public domain) to inform their results, and other articles published in locally refereed scientific journals. We made every effort to be as comprehensive as possible, but it is conceivable that a few published papers meeting our criteria for inclusion may have been missed by our search process. Nevertheless, our objective was primarily focused on capturing and characterizing the general trends and overall patterns, which may be inferred from appropriately, ethically, and transparently communicated (i.e., subject to peer-review, properly documented, accessibly archived, and demonstrably repeatable) scientific research in topics relating to the biology of Philippine amphibians and reptiles.

For each article, we extracted the following information to determine trends and patterns in Philippine herpetological research: (1) year of publication, (2) major focal taxon (amphibians [frogs/toads or caecilians], lizards, snakes, crocodiles, turtles, and a combined category “multiple taxa” for studies involving combinations of each major group), and each paper’s (3) research theme as discussed above (see Table 1). Then, we performed a Poisson regression and local polynomial regression to test for a trend in the number of papers published during the last two decades. Because we wanted to address the technological advances of the last quarter century, we characterized the variable categories of evidence, employed by papers that included new species descriptions (see also Brown and Stuart 2012). We achieved this by classifying every taxonomic publication according to the type of data that was used (e.g., morphological, bioacoustic, molecular, and/or combinations of these data).

**Table 1.** The categories used to identify types of peer-reviewed papers involving Philippine herpetology published from 2002–2022 (based on the five topical themes of Brown et al. 2002a).

Category	Description
Diversity and distribution	surveys, checklists, distributions (range extensions, new island records), and measures of diversity
Taxonomy	species descriptions and taxonomic revisions
Ecology	natural history, community ecology, and population biology
Phylogenetics and biogeography	phylogenetics (excluding new species description), evolutionary biology, and biogeography
Conservation	conservation, outreach, threats, and methods

### ﻿Geographical patterns

Available geographic coordinates were extracted from articles focusing on new species descriptions, species distribution records, natural history notes, and targeted herpetological inventories. To assess country-wide geographical patterns of published herpetological studies from the literature, we projected occurrence records on a map of the archipelago and georeferenced all points by referring to museum records and biodiversity information resources (below), if necessary. For articles that did not report geographic coordinates but which did include specific locality information (e.g., island, province, municipality, barangay, or other unique identifiers), we georeferenced occurrence data in Quantum GIS v. 3.22 using the Philippine gazetteer available from DIV-GIS Database (https://www.diva-gis.org/). We included regions, areas, or single sites, which have been recently surveyed, and for which all specimen-associated data have been properly curated (e.g., in museum databases linked to accessible voucher specimens, ensuring repeatability and transparency; and from the community of the biodiversity repository institutions which provide unrestricted access to specimen-associated data) and published in accessible, publicly available databases such as the Global Biodiversity Information Facility (GBIF; https://www.gbif.org/), VertNet (http://vertnet.org/), and iDigBio (https://www.idigbio.org).

Our presentation of geographical patterns made use of the last half-century’s prevailing context for biogeographical studies in the archipelago, namely the Pleistocene Aggregate Island Complexes (PAIC) model (Brown and Diesmos 2002; Brown and Guttman 2002; Brown et al. 2013a). This model attempts to capture the biogeographic terrestrial subregions of the archipelago based on earlier studies that traced underwater bathymetric contours around the archipelago’s major landmasses (Inger 1954; Leviton 1963a; Brown and Alcala 1970a), so as to reflect patterns of island amalgamations, terrestrial connections involving major landmasses and adjacent island banks, and other exposure of land caused by oscillating Pleistocene sea levels (review: Brown et al. 2013a). Together, the frequently discussed “PAIC Paradigm” illustrates the locations of simplified, reasonably accurate, inferred land connections that may have partitioned and isolated terrestrial vertebrates into the variable and distinct faunal compositions we find today, on the islands which together make up each unique faunal subregion: The Luzon PAIC, and those of Mindanao, Mindoro, West Visayan (also referred to as Negros-Panay), Palawan, and the smaller Romblon Island Group (RIG), the Sulu Island Group (SIG) (Fig. 1), and a few small islands which are associated with larger landmasses but which were never fully connected to them. This latter group includes Siquijor Island, adjacent to the West Visayan PAIC; Camiguin Sur Island, adjacent to the Mindanao PAIC); and finally, isolated island groups that form minor but unique subcenters of biodiversity, but which are not part of or strongly associated with, the major PAICs (e.g., Babuyan and Batanes Island Groups, north of Luzon).

### ﻿First authorship nationality and gender gaps

Finally, as a simplified but important general first step towards exploring disparity and gender gaps in Philippine herpetology, we also determined the nationality (Filipino vs all non-Filipino nationalities/“foreign”) and traditional biological gender (male vs female) of the first author of each article. Although we feel this somewhat crude, excessively binary view will not capture nuances needed to truly assess equity gaps in Philippine herpetology, we consider it a first step and a point from which we hope future discussions and steps towards broadening engagement can begin (see Ramírez-Castañeda et al. 2022). In particular, we emphasize that our use of the term “gender” and its binary assignment in this preliminary analysis is used solely for the purposes of assessing gender gaps in Philippine herpetological research and does not imply a binary nature of the term (see Rock et al. 2021). All analyses and visualizations were performed in R Studio v. 4.2.2 (R Core Team 2022). R code and documentation (R markdown HTML, Suppl. materials 1, 2) are available on GitHub (https://github.com/csupsup/PhilHerpsRev).

## ﻿Results

**Figure 2.**
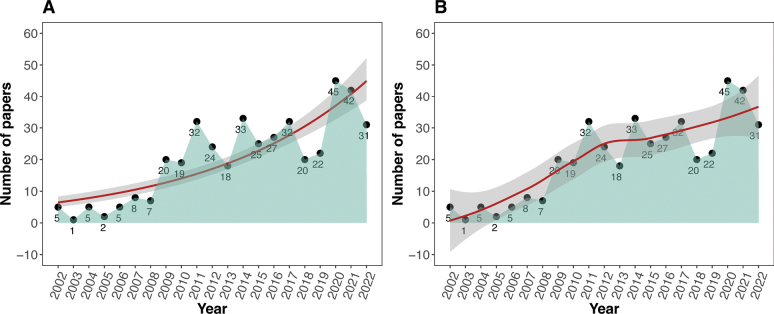
Philippine herpetological research papers published (2002–2022) **A** with Poisson regression line and **B** local polynomial regression. The gray shading represents the 95% confidence interval of the regression models, and the light green shade indicates the trend in terms of the number of papers published per year.

We compiled a total of 423 peer-reviewed scientific articles on Philippine herpetology, published from 2002–2022 (see Suppl. material 1). There was a significant increase in publications during the years based on our Poisson regression analysis (*X^2^*_(1, *n* = 21)_ = 131.9, *p* < 0.001). Approximately four publications per year, for the majority of the first decade (2002–2008), was a general trend that increased sharply to approximately 28 publications per year across the second decade (2009–2022; Fig. 2). The years with the highest number of publications were all during the past 11 years: 2020 (*n* = 47), 2021 (*n* = 42), 2014 (*n* = 32), 2011 (*n* = 31), and 2022 (*n* = 31). Notably, this dramatic seven-fold increase in publication rates during the last 11 years, comprises a remarkable 83.4% of all publications in Philippine herpetology since 2002.

### ﻿Research themes

**Figure 3.**
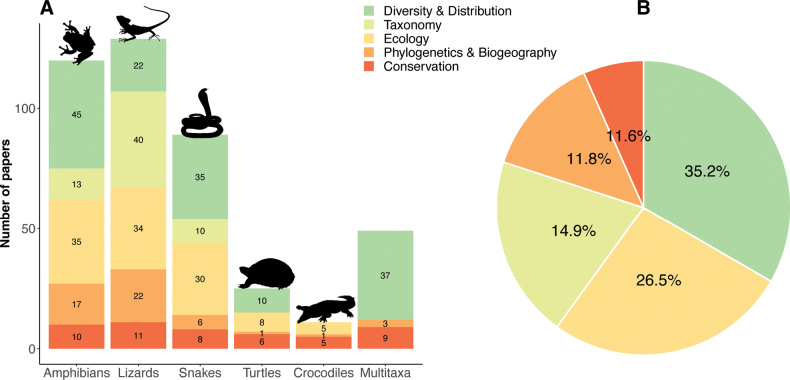
Total of published papers compiled (2002–2022) **A** category per taxon and **B** category proportions in percentages.

Of the 423 articles we reviewed, half the papers were conducted primarily on reptiles, with lizards and snakes comprising nearly equivalent proportions of the total, or 30.5% (*n* = 129), and 21% (*n* = 89), respectively (Fig. 3A). Amphibians comprised 28.4% (*n* = 120), turtles 5.9% (*n* = 25), and crocodiles 2.6% (*n* = 11). The remaining papers involved multiple taxa (11.6%, *n* = 49), with the predominant combinations of taxa most often employed by regional faunal studies: including all amphibian and reptile species recorded for a given island, region, local area, or specific site (Fig. 3A). Of these same 423 papers, 35.2% fell under the category Diversity and Distribution (*n* = 149), 26.5% were focused on Ecology (*n* = 112), 14.9% constituted Taxonomy (*n* = 63), 11.8% were classified as Phylogenetics and Biogeography (*n* = 50), and 11.6% were in the Conservation (*n* = 49) category (Fig. 3B).

A quarter of the Diversity and Distribution studies were targeted toward herpetological inventories and surveys (multiple taxa, 25.8%), while the remaining constituted brief distribution reports on amphibians (30.2%) and reptiles (45%), most of which consisted of single occurrence records, provided for a single species, not previously reported for that island, region, or province. Studies in Ecology, including short natural history notes, were almost equally represented among amphibians (31.2%), lizards (20.4%), and snakes (26.8%) but with few papers on turtles (7.1%) and crocodiles (4.5%). Notably, most publications in taxonomy during the last two decades focused on lizards (63.5%), with amphibians and snakes comprising 20.6% and 15.9%, respectively. Similarly, Phylogenetics and Biogeography publications primarily were conducted on lizards (44%), followed by amphibians (34%), and snakes (12%). All taxa were represented well in Conservation studies (10–20%).

### ﻿New species descriptions

**Figure 4.**
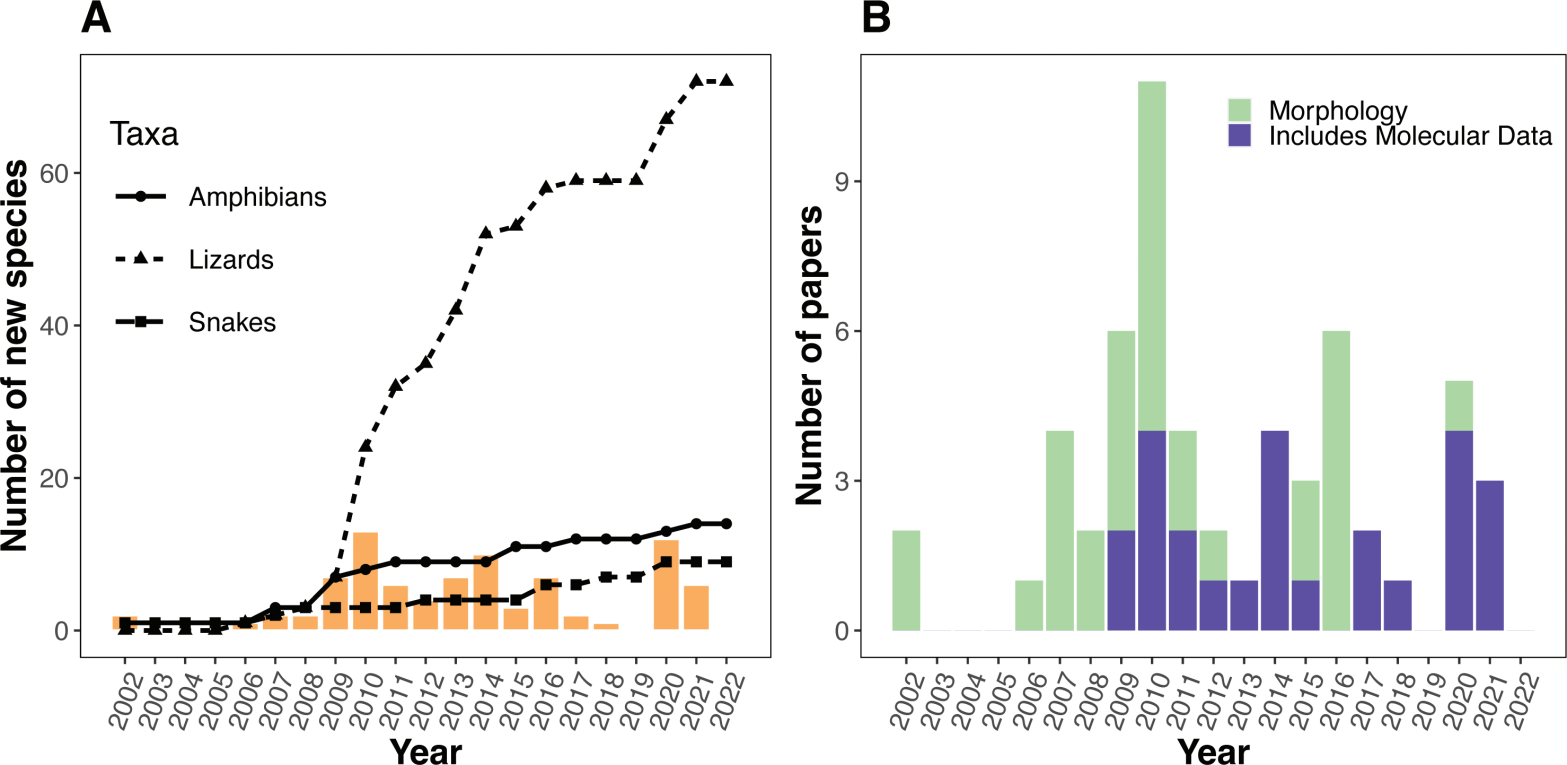
**A** Accumulation curves depicting the relationships between the cumulative total number of species per taxon (key) and the year of description. The orange bars indicate the number of new species described annually **B** number of papers published under the Taxonomy category, grouped by data type.

**Figure 5.**
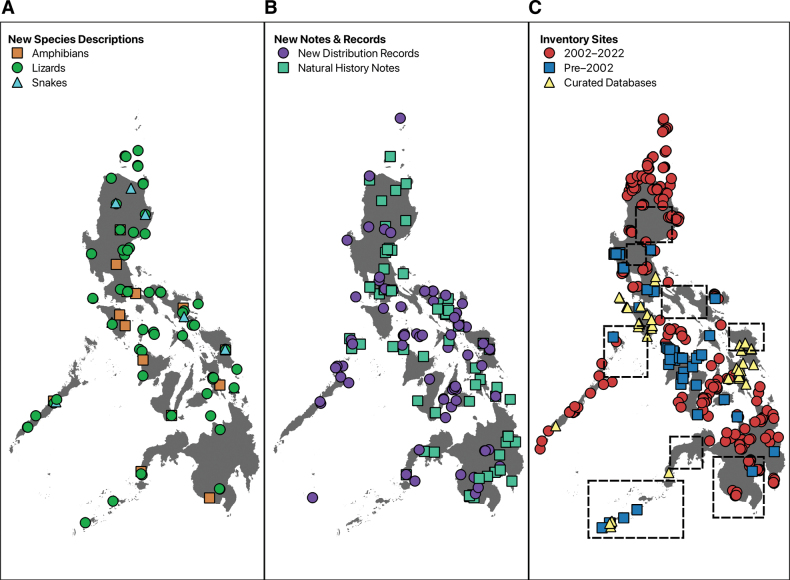
Maps showing the type localities of **A** new species described in the past two decades **B** localities of new distribution records and natural history notes, and **C** the inventory sites available from publications and curated databases. Dashed squares indicate understudied regions (**C**) where comprehensive surveys have not yet been published, but natural history notes and new geographical records have begun to fill spatial gaps.

A total of 95 herpetological species (72 lizards, 14 amphibians, and 9 snakes) was reported as new to science and formally described during a prolific period of taxonomic activity spanning the last two decades (Fig. 4A). New species descriptions started to increase in 2009 with peaks in 2010 (18 new species), 2014 (10 new species), and 2020 (11 new species). Species description papers employing only morphological data were published more frequently between 2000 and 2012 but continue to decrease until the present time. Although the first use of molecular data (primarily mitochondrial DNA sequences) in systematic and biogeographical studies of Philippine herpetofauna occurred earlier, the use of molecular data as a form of evidence to justify new species recognition in taxonomic publications first began as a practice in 2009 and steadily increased, eventually becoming nearly (but not quite) routine by 2022 (Fig. 4B). When we mapped the geographical position of origin for holotype specimens of these new species (see Suppl. material 2), we found that most of the holotypes originated on Luzon PAIC islands (43.2%) or were from the Mindanao PAIC landmasses (22.1%), whereas few came from the Palawan PAIC (9.5%), the West Visayan PAIC (8.4%), Mindoro Island/PAIC (7.4%), small islands of Romblon Province (RIG; 3.2%), the Sulu Archipelago (SIG; 2.1%), the Babuyan and/or Batanes groups of islands (2.1%) and the isolated island of Camiguin Sur (2.1%) (Fig. 5A; total number of points = 95).

### ﻿Geographical patterns

Brief distribution records, focused on single occurrences for single species from areas where they had not previously been reported were published primarily in one journal (Herpetological Review), on the basis of records from the PAICs of Mindanao (31.5%), Luzon (27%), and the West Visayas (16.9%) (Fig. 5B; total number of points = 89; see Suppl. material 2). The majority of new distribution records from the Mindanao PAIC were from the central and southern regions of this large island, with few notes reporting occurrences of particular species from the eastern and western regions of Mindanao or from the northern Mindanao PAIC islands of Samar, Leyte, and Bohol. In contrast, single species distribution records from the Luzon PAIC were not grouped geographically and were more randomly distributed (Fig. 5B). On the West Visayan PAIC, the islands of Cebu, Negros, and Masbate were the focus of most new geographical distribution records. Records from Palawan (11.2%) primarily were from the northernmost portion of this island, as well as the Calamian group of islands, to its north. The remaining distribution records originated in the RIG (6.7%), SIG (2.2%), Mindoro Island/PAIC (2.2%), the Batanes Island Group (1.1%), and Siquijor (1.1%). Occurrence points from natural history notes (Fig. 5B; total number of points = 83) were biased towards the two largest PAICs, Luzon (45.7%) and Mindanao (37.3%).

Targeted herpetological surveys or sustained site-based studies attempting to characterize comprehensively, whole island communities, or regional faunas were concentrated during the last 20 years most heavily on the Luzon (35.6%) and Mindanao (33.1%) PAICs (Fig. 5C; total number of points = 247). Within the Luzon PAIC, targeted fieldwork was conducted mostly in several more northern regions of this large island, but a few sites were sampled well, along southern Luzon’s Bicol Peninsula. Within the Mindanao PAIC, surveys were conducted primarily in the northern and northeastern regions (including several large mountains on northern Mindanao Island itself, but also on the East Visayan islands of Samar and Leyte). Only a relatively few sites have been sampled reasonably well in the southern portion of Mindanao Island during the last two decades. The West Visayan PAIC received attention in the form of a few surveys (10.1%) in recent years, most notably on Cebu, Panay, and Negros Islands. Approximately equal numbers of survey points were collated from the RIG (5.6%), Palawan (4.8%), and the island groups of Babuyan (5.3%) and Batanes (3.2%). There were also survey efforts in Camiguin Sur (1.6%) and Siquijor (0.4%).

### ﻿First authorship nationality and gender gaps

**Figure 6.**
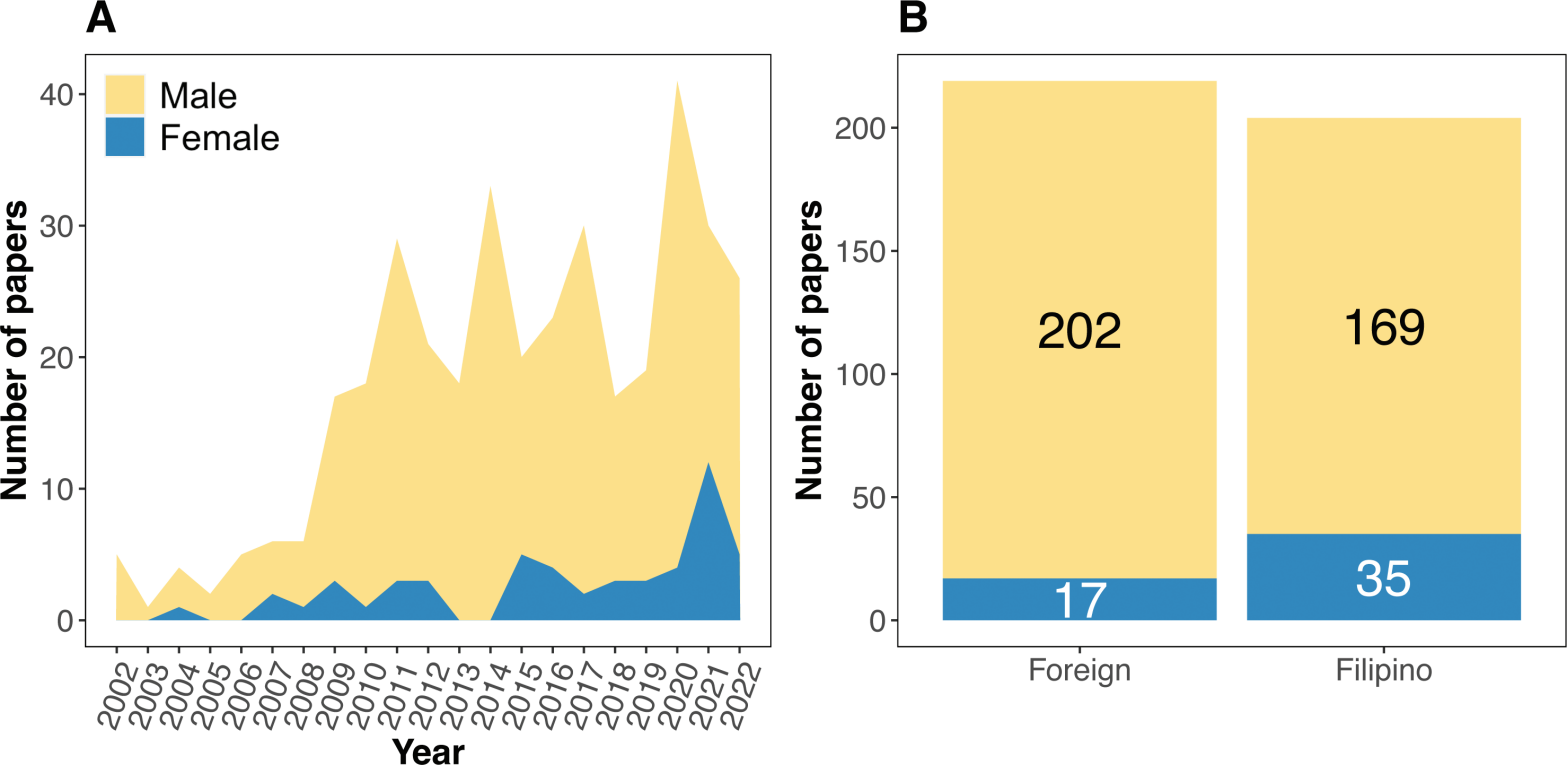
The total number of papers grouped by the first author’s **A** gender (7.13 male: 1.0 female) and **B** nationality (1.07 foreign: 1.0 Filipino).

We found approximately equal cases of Filipino- versus foreign-first authorships (1.0:1.1), with the number of Filipino first authors steadily increasing during the period of 2002–2022 (Fig. 6A). In the last decade (2013–2022), 63.7% of papers were first-authored by Filipinos which is a three-fold increase from the 2002–2012 estimate (21.1%). We identified a wide gender gap in Philippine herpetology, with males outnumbering females when quantified as a function of first authorship (7.1:1.0) during the past 20 years; only approximately 12.3% of all papers were first-authored by females (Fig. 6B).

## ﻿Discussion

We are greatly encouraged by the increasingly collaborative and much more equitable and inclusive nature of Philippine herpetology. Great strides have been made during the past two decades in terms of our collective knowledge of herpetological biodiversity in the archipelago, public awareness and interest in amphibians and reptiles, and an increasingly vocal environmentalist movement–combined with the establishment of new protected areas and new laws for protecting historically underappreciated groups such as amphibians and reptiles (Diesmos and Brown 2011; Brown et al. 2012a; Diesmos et al. 2014; Gonzalez et al. 2018). The past 20 years provide us with a clearer picture of the progress made, gaps filled and unfilled, and collaborations formed, which now serve as a springboard for or guide to additional progress in the coming decade for our community.

### ﻿Herpetology studies during the last two decades

Our appreciation of the diversity and distribution of amphibians and reptiles in the Philippines has increased during the last 20 years, with the continued efforts towards conducting targeted faunal inventories and resurveys, supplemented by short distribution notes reporting single species occurrences (Brown et al. 2012a, 2013a). Faunal inventories have dominated herpetological studies during the last two decades, which is beneficial for informing habitat conservation measures (Margules and Pressey 2000) and these works positively impact species conservation assessments (see Gonzalez et al. 2018; IUCN 2022). Importantly, data from these studies help address knowledge gaps in species distributions, broaden stakeholder inclusion and participation in Philippine herpetology, and improve our collective knowledge and understanding of biodiversity (Hortal et al. 2015).

Region-wide herpetological inventories in recent years covered a wide range of habitats and utilized multiple datasets like field records and museum specimens (Oliveros et al. 2011; Siler et al. 2011d, 2012b; Brown et al. 2012a, 2013b; Devan-Song and Brown 2012; Mcleod et al. 2012; Sanguila et al. 2016; Supsup et al. 2016). Complementing these regional inventories were site-based studies along different environmental gradients in inland montane habitats (Alcala et al. 2004; Plaza and Sanguila 2015; Supsup et al. 2017, 2020; Gojo-Cruz et al. 2018; Pitogo et al. 2021a; Sanguila et al. 2021) and island ecosystems (Bucol et al. 2011; Siler et al. 2012a; Venturina et al. 2020; Clores et al. 2021; Meneses et al. 2022). Species accounts resulting from these targeted surveys provide a much clearer picture of herpetological communities occurring in several critically threatened habitats, which serve as important baseline studies to track long-term changes in amphibian and reptile communities over time (e.g., Brown et al. 2000a, vs Siler et al. 2011d; Siler et al. 2012b, vs Meneses et al. 2022; Brown and Alcala 1986, vs Supsup et al. 2016; Brown 2015, vs Pitogo et al. 2021a). Continued conducting of targeted surveys and resurveys are still urgently needed to gather empirical field data and species occurrences throughout the Philippines, especially in understudied regions, small islands (see discussion on geographical patterns), and other threatened habitats (e.g., karst habitats, lowland forest fragments, and understudied protected areas). Repeated resurveys spanning time, seasonal variation, space, and disturbance (both anthropogenic [deforestation, mining, road-building] and natural [typhoons, earthquakes, tsunamis]; Peterson et al. 2017) in key areas and habitat types, whenever resources are available, are strongly recommended (Brown et al. 2012a; Sanguila et al. 2016).

Many of the ecology studies of Philippine herpetofauna are short observations on species’ reproductive biology, natural history, habitat use, diet, and behavior (see Suppl. material 1); all of these biodiversity information products supplement our limited understanding of the natural history of many endemic and native species (i.e., Meneses 2020; Brown et al. 2021a, b, c; Maglangit et al. 2021; Pitogo 2021). However, more in-depth ecological and herpetological community composition studies have bolstered patterns that were apparent two decades ago (Brown et al. 2002a) and revealed new trends. Elevational studies have repeatedly characterized a positive relationship between elevation and proportional endemism (the portion of species at a given site that is endemic to the Philippines), but an inversely proportional relationship between elevation and species diversity (Brown and Alcala 1961; Brown et al. 2000a; Ferner et al. 2001; Diesmos et al. 2004a; Siler et al. 2011d; Brown et al. 2013a; Pitogo et al. 2021b; Brown et al. 2022; Meneses et al. 2022), with a potential mid-elevation peak in species diversity was detected in some areas (Gojo-Cruz et al. 2019; Supsup et al. 2022). There is evidence that elevation strongly shapes broad-scale distribution patterns of Philippine herpetological communities, but other habitat characteristics were also found to influence the fine-scale distribution of species (Siler et al. 2011c; Pitogo et al. 2021b; Seidl et al. 2019; Supsup et al. 2020, 2022). Functional traits potentially drive distribution patterns (Pitogo et al. 2021b) and may influence species’ responses to environmental changes (Scheffers et al. 2013). Resource partitioning has also been recently investigated in amphibians (Shooman-Goodier et al. 2019; Plaza et al. 2021) and reptiles (Garcia et al. 2014; see also Auffenberg and Auffenberg 1988), improving our knowledge of trophic interactions among co-occurring species. These hypothesis-driven studies are welcomed developments in Philippine herpetology and should pave the way for a greater understanding of herpetological communities, limiting resources, and habitats critical for individual species’ persistence and community resilience. Despite advances in research focusing on community ecology, there has been limited progress in studies involving reproductive biology, development (see Flores et al. 2023), physiology, and behavior (Lama and Senarillos 2023) during the past 20 years–as was also the case during the first review (Brown et al. 2002a).

Complementing field-based studies are works utilizing genetic data to understand the evolutionary history and phylogenetic relationships of species. The subdisciplines of model-based statistical phylogenetic systematics (Hillis et al. 1996) and quantitative biogeographical inference (Ronquist 1997) were just emerging at the time of the first review (Brown et al. 2002a). During the last two decades, numerous studies involving robust genetic datasets from Philippine species (and/or whole clades), from multilocus Sanger sequence datasets to genome-scale datasets, orders of magnitude more expansive, have provided inference into biogeographic patterns and a diversity of underlying processes. These studies have contributed greatly to our appreciation of true species diversity, species’ distributions, routes of colonization, and the dynamic geographical template’s role in contributing to evolutionary diversification within the archipelago (Brown and Diesmos 2009; Blackburn et al. 2010, 2013; Siler et al. 2012a, 2014a, b, c; Brown et al. 2013a, 2015a, 2016; Linkem et al. 2013a; Oaks et al. 2013, 2019, 2022; Brown 2016; Chan and Brown 2017). These studies also provide insights into surprising and intriguing evolutionary relationships not observed in other vertebrate groups (Weinell and Brown 2017; Meneses et al. 2020a; Weinell et al. 2020b), and patterns of cryptic speciation (Sanguila et al. 2011; Barley et al. 2013, 2021; Linkem and Brown 2013; Welton et al. 2013a, 2017; Weinell et al. 2020a, c; Abraham et al. 2021; Chan et al. 2021), many of which have informed taxonomic developments, fueling the description of nearly 100 new species during the past twenty years (see Taxonomic Progress, below). We expect an increase in this type of work in coming years, especially with the widespread availability and declining cost of genomic data (Carter et al. 2023), which increases accuracy in phylogenetic inference, while greatly clarifying previously obfuscated species boundaries by allowing nuanced insight into related phenomena, such as gene flow, admixture, hybridization, lineage sorting, and retention of ancestral polymorphism (Alexander et al. 2016; Wood et al. 2020; Chan et al. 2021).

### ﻿Conservation of Philippine herpetofauna

The last two decades involved increasing numbers of conservation studies, particularly focused on large and charismatic species like crocodiles (van de Ven et al. 2009; van der Ploeg et al. 2011; Brown et al. 2021a), turtles (Abreo et al. 2016; Sy et al. 2020), monitor lizards (Welton et al. 2013b, 2020; Abaño-Sarigumba et al. 2018), and sailfin lizards (Siler et al. 2014d; Heinrich et al. 2021). These species are most often the highly traded and threatened species in the Philippines (Cruz and Lagunzad 2021). Although amphibians receive less attention in the conservation literature, several efforts were made to highlight their high vulnerability to environmental perturbations and climatic changes (Alcala et al. 2004, 2012a, b; Brown et al. 2012a; Diesmos 2012; Diesmos et al. 2014). Recent amphibian rediscoveries of “lost” species have captured public attention (e.g., Bittel 2015; Pitogo and Saavedra 2021), which bolsters public interest and stimulates conservation research and action in overlooked taxa (e.g., Brown and Alcala 2000; Pitogo and Saavedra 2023). Overall, peer-reviewed literature on the conservation of Philippine herpetofauna has increased substantially from 20 years ago (Brown et al. 2002a). We are encouraged by these developments and hope to maintain this momentum, build further on these gains (including the development of more outreach materials in support of conservation objectives), and strive to improve the conservation attention to Philippine amphibians and reptiles (Brown et al. 2012a; Gonzalez et al. 2018).

Based on the most recent and updated IUCN assessments on Philippine herpetofauna (ca. 475 species), approximately 13.2% (63 species) and 13% (62 species) are threatened and data deficient, respectively (IUCN 2023). Upwards of 5.9% (28 species) are still unassessed. Notably, 131 of these 153 species are Philippine endemics, many of which have not been observed since their original descriptions. Many additional unrecognized and unprotected species await taxonomic descriptions and, as such, are at increased risk of extinction (McDonald et al. 2022). Continued lowland habitat loss, brought about by forest conversion into less complex habitats that are not suitable to support high levels of biodiversity is likely the most substantial threat to these taxa (Heaney and Regalado 1998; Brown et al. 2002a; Diesmos et al. 2004a; Suarez and Sajise 2010; BMB-DENR 2016; Gojo-Cruz et al. 2019; Decena et al. 2020; Supsup et al. 2020). Although there has been an increase in the establishment of protected areas to avert the threat of habitat loss, many of these protected areas (PAs; i.e., national parks, natural parks, biotic areas, protected landscapes) do not overlap with key biodiversity areas (Mallari et al. 2015; Supsup et al. 2023), have poor to fair management effectiveness (Madarang et al. 2017), have never been properly inventoried for terrestrial biodiversity despite their establishment decades ago (Brown et al. 2002b), and can only marginally reduce forest cover loss (Apan et al. 2017). Despite these challenges, we acknowledge that habitat protection through the establishment of PAs and other effective area-based conservation measures are the first steps and are still the most effective measures for long-term species conservation and survival/persistence. Additionally, public education and societal awareness concerning threats posed by emerging infectious diseases (Swei et al. 2011a, b; Diesmos et al. 2012; O’Hanlon et al. 2018; Byrne et al. 2019) and pet-trade facilitated introductions of highly competitive alien and invasive species (Diesmos et al. 2006; Pili et al. 2019, 2021) are all on the rise.

### ﻿Taxonomic progress

At the time that the state of Philippine herpetology was last reviewed, Brown et al. (2002a) recognized a total of 101 species of Philippine amphibians (77% endemic) and approximately 258 species of Philippine reptiles (65% endemic). During the last 20 years, during which nearly 100 new species were added to the archipelago’s fauna, we recorded differing, taxonomically dependent proportional increases in the number of additionally recognized reptiles (258 vs 361, 28%) versus amphibians (101 vs 114, an 11% increase) between 2002 and 2022 (Fig. 3A), indicating that the archipelago’s native species diversity is far from comprehensively understood. This taxonomic shortfall warrants continued surveys and resurveys, plus targeted taxonomic revisionary attention to particular clades before we might conclude that the fauna is reasonably well characterized. As such, we can anticipate additional increases in cumulative total species diversity estimates in the coming decades, but the question remains, by how much?

Based on the numbers of suspected undescribed species known at that time, Brown et al. (2002a) suggested that herpetological species diversity might increase from 101 to possibly as many as 125–130 (~ 22%) amphibians and from 258 to approximately 275–280 (~ 8%) reptiles. Explanations for why amphibian diversity proportionally increased by less (11%) than the 22% estimated by Brown et al. (2002a) or why reptiles species diversity increased by proportionally far more (28%) than the 8% estimated by Brown et al. (2002a) appears to be a simple function of the fact that researchers (who were actively completing taxonomic studies) showed more interest in systematic and biogeographic questions for which Philippine reptiles represented preferable focal study subjects (e.g., Siler et al. 2009a, 2010a, b, c, d, 2011a, b, c, 2012a, 2014a, b, c, e; Welton et al. 2009, 2010a, b; Brown et al. 2010a, b, c; Linkem et al. 2010a, b, c, 2013; Barley et al. 2013, 2020, 2021; Weinell et al. 2020a, b, c; Eliades et al. 2021) than was the case for research topics involving amphibian study systems (Brown et al. 2009, 2015a, b; Siler et al. 2009b, c, 2010e; Fuiten et al. 2011; Brown 2015; Diesmos et al. 2020; Herr et al. 2021).

Recent efforts to conduct comprehensive herpetological surveys and resurveys have provided a near-complete estimation of the amphibian and reptile diversity and endemism of several islands (e.g., Siler et al. 2012b vs Meneses et al. 2022), mountain ranges (Brown et al. 2000a vs Siler et al. 2011d), or other conspicuous geographical subcenters of diversity in the archipelago through the years (Plaza and Sanguila 2015; Sanguila et al. 2016, 2021). The notable progress of an increased number of described species has been species descriptions (Brown et al. 2010a, c, 2011a, b; Brown 2015; Barley et al. 2021), resurrections of species (e.g., Brown et al. 2000b; Siler et al. 2020), redescriptions of poorly understood taxa (Davis et al. 2014, 2015; Wynn et al. 2016), and revisionary considerations of species boundaries within entire clades (e.g., Linkem and Brown 2013; Brown et al. 2015a, b; Barley et al. 2020). The vast majority of these studies involved the integration of traditional morphological characters (typical of the early 2000s; Brown and Stuart 2012) with molecular data, bioacoustic analyses, ecological information, or other independent data streams (Fig. 4B).

The majority of the 95 species newly described or recognized during the last twenty years are lizards (Fig. 3A); these are dominated by new species of the genera *Brachymeles* (*n* = 12) and *Pseudogekko* (*n* = 5; see Siler at al. 2020; Brown et al. 2020). The genus *Parvoscincus* was expanded by 13 species (Brown et al. 2010c; Linkem and Brown 2013; Siler et al. 2014e) and the genus *Eutropis* by nine new taxa (Barley et al. 2020, 2021). Other new lizard species were described from the genera *Lepidodactylus*, *Gekko*, *Luperosaurus*, *Varanus*, *Cyrtodactylus*, and *Lygosoma*; in total, more than 72 previously unrecognized lizard species have been identified in the past two decades. In many ways, the most spectacular lizard discovery of the past two decades involved the description of a third species of frugivorous monitor lizard, *Varanusbitatawa* from the southern Sierra Madre Mountain Range of Luzon Island (Welton et al. 2010c, 2012), which has since been confirmed from northern Luzon, including the northernmost reaches of the Cordillera Mountain Range or western Luzon (Abaño-Sarigumba et al. 2018; Meneses et al. 2020b). Five other species of Philippine-endemic monitor lizards in the genus *Varanus* were described during the last twenty years as well (Koch et al. 2010; Welton et al. 2014b).

In amphibians, the greatest taxonomic activities were associated with seven descriptions of new Ceratobatrachid frogs in the genus *Platymantis* (Siler et al. 2007, 2009a, 2010e; Brown et al. 2015a, b; Diesmos et al. 2020). Additional discoveries and descriptions of new species were assigned to the genera *Limnonectes*, *Sanguirana*, *Pulchrana*, *Leptobrachium*, and *Kaloula* (Brown et al. 2009a, 2016, 2017; Siler et al. 2009b; Fuiten et al. 2011; Abraham et al. 2021; Herr et al. 2021). These recorded a total increase of 14 amphibian species in two decades, and we suspect that many more await discovery, because many amphibian clades are in urgent need of taxonomic resolution (e.g., *Platymantis*, *Philautus*, and *Occidozyga*; Brown et al. 2015a, Chan et al. 2021, 2022; Flury et al. 2021), which is currently challenged by limited numbers of recordings (insufficient sample sizes necessary to permit quantitative analyses and statistical tests for species delimitation) or a complete lack of acoustic data for particular species or species groups (Brown and Alcala 1994; Alcala and Brown 1999; Hertwig et al. 2011; Herr et al. in press; Khalighifar et al. 2021).

One notable and striking recent discovery was the characterization of an ancient, archipelago-wide clade–a nearly 40 million-year-old endemic Philippine evolutionary radiation, now recognized as the archipelago’s only endemic reptile family: snakes of the clade Cyclocoridae (Weinell and Brown 2017). Two surprising elements of this discovery were apparent; first, the unpredicted finding that snakes of the genera *Cyclocorus*, *Hologerrhum*, *Oxyrhabdium*, and *Myersophis* were a monophyletic group (they had previously and variably been treated as members of separate families or left incertae sedis, of unknown taxonomic affinities; McDiarmid et al. 1999). Second, it was clear from multilocus phylogenetic analyses that an unnamed, genus-level lineage had been overlooked (Weinell and Brown 2017; Weinell et al. 2020a). Description of the new miniaturized genus and species of snake of the family Cyclocoridae, *Levitoniusmirus*, was based on three specimens of this secretive, fossorial snake from Samar and Leyte Islands. *Levitonius* exhibits highly distinctive morphology associated with its miniaturized body form, fossorial habitat, and unique diet, consisting solely of soil invertebrates (Weinell et al. 2020a). This discovery also used CT-scan, a novel method to characterize deep internal anatomy, together with molecular data, traditional morphological characters, diet, and ecological niche data (Weinell et al. 2020a), a novel degree of data-type integration to be associated with a taxonomic description for a Philippine species. Subsequent phylogenomic analyses (Das et al. 2023) confirmed the early-branching phylogenetic placement of Cyclocoridae, closely related to the globally distributed snake clade Elapoidea, which includes coral snakes and cobras. Other, highly unique, or unpredicted snake species discovered included a new species of blind snake phenotypically similar to *Acutotyphlops* (Wallach et al. 2007) from northern Luzon and the highly distinctive, krait-like *Calliophissalitan* (Brown et al. 2018, 2021b). The former is a genus otherwise restricted to the Solomon Islands, which creates a conspicuously unusual and disjunct distribution (the single Philippine species has yet to be included in a phylogenetic analysis, which would be necessary to evaluate this disjunct and somewhat suspect taxonomic/geographic placement). In contrast, *Calliophissalitan* is related to the giant, long-glanded tropical coral snakes of the *C.bivirgata* group and most likely constitutes a separate, unique invasion of the archipelago, apart from other Philippine elapid snakes. In summary, nine species of snakes were described or newly recognized, in the genera *Acutotyphlops*, *Calamaria*, *Calliophis*, *Dendrelaphis*, *Hemibungarus*, *Levitonius*, *Malayotyphlops*, and *Lycodon* (Gaulke 2002, 2011; Wallach et al. 2007; Siler et al. 2013; Wynn et al. 2016; Weinell and Brown 2017; Leviton et al. 2018; Weinell et al. 2019, 2020a, b, c).

### ﻿Geographical patterns of herpetological surveys

Our synthesis of available geographic data suggests that the last two decades were characterized by a significant surge of herpetological research across the archipelago. Herpetological surveys conducted on most major islands, particularly those that were not visited before (e.g., island groups of Babuyans, Batanes, and Romblon Province) have led to a stunning number of new species discoveries, elevating sharply the herpetological diversity of the country. Rediscoveries of poorly known species have also provided new insights about their population status and ignited the needed hope for conservation (e.g., Diesmos et al. 2004b; Siler et al. 2011b, c; Bittel 2015; Oliver et al. 2020; Supsup and Carestia 2020; Brown et al. 2021b; Pitogo and Saavedra 2021, 2023; Meneses et al. 2022). However, despite the highly celebrated discoveries, much work is still needed because many small islands and isolated habitats remain unexplored or have not been surveyed thoroughly (see Fig. 6). The islands of Jolo and Basilan in the Sulu Archipelago are the notably less explored areas of the country despite their zoogeographic importance (Seale 1917; Taylor 1918; Gaulke 1993, 1994, 1995). Few biologists have visited these islands during the last century due to logistical and security constraints. The only attempt at a comprehensive study of this archipelago (with specimens collected and still available for reconsideration) was the work of Taylor (1918); unfortunately, many of his specimens from the region, including holotypes of several of the Sulu Archipelago’s endemic species (e.g., *Luperosaurusjoloensis*, *Brachymelesvermis*, *B.suluensis*) were lost during World War II (Brown and Rabor 1967; Brown and Alcala 1974; Gaulke 1993, 1994, 1995; Uetz et al. 2023). Because of the lack of studies on these islands, many wildlife biologists and biogeographers are still puzzled by uncertain taxonomic affinities and conservation status of the endemic biodiversity of the Sulu Archipelago (Siler et al. 2012c; Spinks et al. 2012; Brown and Siler 2013; Chan et al. 2021).

The apparent absence of peer-reviewed herpetological studies on the islands of Polillo (east of Luzon) and Siargao Island (northeast of Mindanao) is somewhat artefactual, and due to the fact that the majority of fieldwork conducted on these two small islands is only available as unpublished reports (but see Ross and Lazel 1991; Nuñeza and Galorio 2015; Sanguila et al. 2016; Quibod et al. 2021); nevertheless, some collection information (specimens deposited in accessible biodiversity repositories such as Smithsonian National Museum Natural History [USNM], University of Kansas Natural History Museum [KUNHM], Philippine National Museum of Natural History [PNMNH], and Father Saturnino Urios University [FSUU]) are readily accessible via online biodiversity repositories (e.g., GBIF, iDigBio). In addition to herpetologically unexplored regions of the country, the intact forest habitats of the central Sierra Madre Mountain Range of Luzon (in particular, higher elevations), including the relatively large but fragmented forests to the west have not been thoroughly explored (but see Brown et al. 2000a, 2007, 2010c, 2013b; Siler et al. 2011c; Gojo-Cruz et al. 2018, 2019). Similarly, isolated high-elevation forest habitats in southern Luzon along the borders of Quezon and Bicol Provinces and in Mindoro have not been explored well. Except for the relatively well-explored Caraga region in northeastern Mindanao (Sanguila et al. 2016), many of Mindanao’s forests remain herpetologically underexplored, a condition which has persisted during the last two decades (and last century); this is particularly true of western, central, and southern Mindanao, from Mt. Piapayungan southward to the Mt. Latian complex (Taylor 1921, 1922a, b; Sanguila et al. 2016; Pitogo et al. 2021a; Maglangit et al. 2022). As in the Sulu Archipelago, the limited availability of published results from field studies on Mindanao is due, in part, to logistical and security challenges, as well as a lack of local regional expertise, training, and experience with field-based herpetological inventories (Sanguila et al. 2016; Pitogo et al. 2021a; Pitogo and Saavedra 2021).

Despite these gaps, we should note that during the last two decades, there have been significant field-based efforts focused on survey-resurvey studies at important, formerly incompletely understood areas. Several key studies have revisited areas that were targeted in periods before 2002, with the general goal of reassessing, completing, and/or providing a time series (before and after comparison) to enable a temporal perspective on faunal investigations conducted previously, and in light of deforestation, land use change, and global climate change: Zambales Mountains (Brown et al. 1996; Devan-Song and Brown 2012), the central Sierra Madre Mountains of eastern Luzon (Brown et al. 2000a; Siler et al. 2011d), the northern portions of the Sierra Madre Mountains (Brown et al. 2013b), the northern Cordillera Mountain Range of western Luzon (Diesmos et al. 2004a; Brown et al. 2012b), Panay Island (Ferner et al. 2001; Gaulke 2011), Cebu Island (Brown and Alcala 1986; Supsup et al. 2016), and Negros Island (Brown and Alcala 1955, 1961, 1963, 1986; Alcala 1958; Bucol et al. 2019), as well as recent resurveys focused on reassessments of the faunas of northeastern, central eastern, and southern Mindanao (Sanguila et al. 2016, 2021; Pitogo et al. 2021a; Plaza et al. 2021).

Many additional, earlier faunal studies (published before 2001; other areas that have been surveyed, but as of yet, have not been published) are now urgent priorities for survey-resurvey studies, hopefully in the near future (Fig. 5C). We recommend undertaking this work as soon as possible because such areas may contain critically important populations of endemic species, ‘lost’ species (i.e., species not encountered since their original descriptions and for which holotype specimens were destroyed in World War II), exceedingly rare species, and species awaiting rediscovery and discovery (currently unknown to science), all of which may already be facing cryptic extinction risk brought by habitat degradation and destruction (McDonald et al. 2022). We strongly encourage researchers to consider understudied areas as top priorities for field-based biodiversity research in the coming years (Fig. 5C). Also, for areas that have not been explored due to logistical obstacles and/or security challenges (e.g., the Sulu Archipelago, southern and southwestern Mindanao Island, etc.), we strongly encourage capacity-building activities for local institutions, universities, and other community stakeholders (e.g., training of residents, students, and other community members) to conduct field-based biodiversity research involving amphibians and reptiles. Such an approach is most likely the safest, most cost-effective, and most feasible strategy for moving forward to address geographical hiatuses and knowledge gaps represented by unexplored, politically charged, or otherwise sensitive areas (see Ramírez-Castañeda et al. 2022). Training residents to effectively survey their local biodiversity reduces reliance on foreign institutions (or groups that are not residents of an area), builds the research capacity of stakeholders who may not have had the opportunity to engage in science, maximizes scholarly equity, and increases the potential for local governance and conservation action.

### ﻿Diversity, equity, and inclusion in herpetology research

The rise of Filipino-first authorship in Philippine herpetological studies during the last two decades (Fig. 6B) is an encouraging development since the first review (Brown et al. 2002a). This substantial increase indicates an increased interest among early-career Filipino researchers in the discipline, which formerly was limited to a few Filipinos and their foreign collaborators. It is apparent that a large proportion of the last two decades of Filipino-led studies were field surveys and descriptive studies, whereas the majority of sophisticated studies utilizing genetic data relying on large molecular datasets or genomic analyses were led by non-Filipinos. This disparity reflects the relatively limited capacity for genomics in the Philippines, highlighting the importance of equitable collaborations to ensure skill and technology transfers between local and foreign researchers. A collaborative approach provides opportunities for capacity development for less experienced researchers (e.g., mentorship, writing, decision-making) and may further improve local interest in scientific research (Ramírez-Castañeda et al. 2022). We have seen many multi-national collaborations during the past two decades, which are consequential and contribute to our present understanding and appreciation of Philippine herpetofauna. However, we also acknowledge that access to these opportunities, including advancement in scientific careers, is limited by many socioeconomic factors.

Women are historically underrepresented in herpetology but there has been an increase in female authorship in research on amphibians and reptiles, potentially narrowing the gap if this positive trajectory continues (Rock et al. 2021). Nevertheless, the gender gap in Philippine herpetology (7.13 male per 1.0 female, as first authors) is far from the global average (1.95 male per 1.0 female first authors); ameliorating this disparity will require more representation from women in scientific publications. Despite the substantial gender gap, we are inspired by the continued emergence of next-generation Filipinas and greatly value their contributions to the advancement of the field during the past 20 years. We hope that many additional early-career women researchers, along with other historically underrepresented Filipino groups, will be encouraged to participate in the study of amphibians and reptiles toward a more inclusive and equitable scientific community in the Philippines. Additionally, finding ways to communicate the results of our studies to the public while narrowing the gap between the scientific community and the lay-/citizen science community will be an important step for rendering our science more accessible to the public and policymakers, while bridging the gaps between science and policy (Young et al. 2014; Ramírez-Castañeda et al. 2022).

### ﻿Future directions

Clearly at a crossroads of topical shifts in research themes, increased engagement, equity, inclusion, and representation of diversity in collaborations, Philippine herpetology has undergone a demonstrable maturation since the first review (Brown et al. 2002a). This work sets the stage for what we hope and anticipate will be an increasingly collaborative and inclusive engagement by diverse kinds of herpetologists during the coming several decades, in continuation of the rich history and development of our collective understanding of the amphibians and reptiles in the archipelago. Much progress has been made in some areas (increased general public education and lay-public interest, increased numbers of publications by a broad array of early-career herpetologists, narrowing of the first authorship gap between Filipinos versus foreigners), whereas other equity gaps (e.g., first authorship gender) still remain, requiring increased attention towards fostering diversity and encouraging the engagement of people from a broader array of backgrounds. It is our hope, in compiling this synthesis, that we can challenge the community of individuals, groups, and institutions interested in amphibians and reptiles of the Philippines to pursue some of the conspicuous gaps in research themes identified here and encourage early-career herpetologists to pursue research topics that have advanced in surrounding countries during the last quarter century, but which have not received comparable interest or attention in the Philippines (e.g., amphibian larval biology, developmental studies, etc.). Similarly, historically understudied geographical gaps identified in this review should be viewed as opportunities for increased attention and enhanced collaboration, both among Philippine institutions and between Filipino and foreign herpetologists. The challenge issued by Walter C. Brown a few years before the last review (e.g., ‘The State of Philippine Herpetology;’ Brown et al. 2002a) still stands: “Rather than view Philippine herpetology as something you might be tempted to divide up, why not just see how much you can accomplish, together, in collaboration?” (W. C. Brown to RMB and A. C. Alcala, personal communication 1998). In accordance with this perspective, and in light of the progress made during the last two decades, we are quite sure that the future of Philippine herpetology will profit most from increased engagement, involving a diversity of people, and embracing increasingly broad thematic research questions in collaboration, all with the common goal of understanding, appreciating, and conserving the archipelago’s spectacularly unique amphibian and reptile fauna.

## ﻿Additional information

### Author ORCIDs

Camila G. Meneses https://orcid.org/0000-0003-0594-5436

Kier Mitchel E. Pitogo https://orcid.org/0000-0002-5785-8281

Christian E. Supsup https://orcid.org/0000-0001-5176-8181

Rafe M. Brown https://orcid.org/0000-0001-5338-0658

### Data availability

All of the data that support the findings of this study are available in the main text or Supplementary Information.

## Supplementary materials

Supplementary material 1 Scientific articles on Philippine herpetology, published from 2002–2022 Data type xlsx https://binary.pensoft.net/file/975760This dataset is made available under the Open Database License (http://opendatacommons.org/licenses/odbl/1.0/). The Open Database License (ODbL) is a license agreement intended to allow users to freely share, modify, and use this Dataset while maintaining this same freedom for others, provided that the original source and author(s) are credited.Camila G. Meneses, Kier Mitchel E. Pitogo, Christian E. Supsup, Rafe M. Brown

Supplementary material 2 Brief distribution records Data type xlsx https://binary.pensoft.net/file/975761This dataset is made available under the Open Database License (http://opendatacommons.org/licenses/odbl/1.0/). The Open Database License (ODbL) is a license agreement intended to allow users to freely share, modify, and use this Dataset while maintaining this same freedom for others, provided that the original source and author(s) are credited.Camila G. Meneses, Kier Mitchel E. Pitogo, Christian E. Supsup, Rafe M. Brown
